# Post-market quality assessment of 22 ciprofloxacin brands by HPLC available in Bangladesh market

**DOI:** 10.1016/j.heliyon.2023.e17180

**Published:** 2023-06-12

**Authors:** Md Hasanuzzaman Shohag, Syed Abdul Kuddus, Esfat M. Saim Brishty, Salman Sakir Chowdhury, Md Tofazzal Hossain, Maruf Hasan, Sabrin Islam Khan, Murad Hossain, Hasan Mahmud Reza

**Affiliations:** Department of Pharmaceutical Sciences, School of Health & Life Sciences, North South University, Dhaka, Bangladesh

**Keywords:** Ciprofloxacin, Tablet, HPLC, Post-market analysis, Bangladesh

## Abstract

Antibiotic resistance has been recognized as a public health threat in recent years, and mortality due to resistance is increasing alarmingly every year. Antibiotic resistance, among many factors, may arise due to the consumption of substandard antibiotic brands that provide subnormal levels of the drug in the blood. Post-market evaluation can provide important information in assessing pharmaceutical products in terms of quality, purity, and therapeutic aspects. Ciprofloxacin, a broad-spectrum antibiotic, has been used against a wide range of infectious diseases in Bangladesh. The present study aimed to determine the quality attributes of twenty-two commonly prescribed brands of ciprofloxacin 500 mg tablet collected from Dhaka city and the rural regions of Jessore. RP-HPLC coupled with UV–visible spectrophotometry was used to determine the potency of ciprofloxacin in tablets, and the zone of inhibition was determined using Kirby-Bauer's disc diffusion method to assess the antimicrobial efficacy against different strains of microorganisms. We found that 95.45% of brands (21 out of 22 brands) of ciprofloxacin tablets met United States Pharmacopoeia (USP) and British Pharmacopoeia (BP) specified potency, whereas one brand failed. From dissolution studies, we observed that 68.2% of brands (15 out of 22 brands) followed USP/NF dissolution test specifications, whereas 31.8% (7 out of 22 brands) failed to release 80% of the labeled amount of drug within 30 min. Drug release kinetics data showed that most brands followed the Weibull drug release kinetic model. Fit factor analysis exhibited that 8 brands out of 22 (36.4%) failed to comply similar dissolution profiles with the reference product. Minimum inhibitory concentrations, assessed against five bacterial strains, further showed good antimicrobial sensitivity by all brands.

## 1 Introduction

Antibiotic resistance is one of the most significant threats to global health. Low & middle-income countries are substantial contributors to antibiotic resistance due to inadequate healthcare standards, consumption of substandard drugs, irrational antibiotic use, poor patient compliance, etc. [1,2]. Bangladesh, a developing country in Asia, demonstrated a high rate of antibiotic resistance in recent years, and the resistance-induced mortality rate elevated drastically [3]. Ensuring the quality of antibiotics, rational application, and proper surveillance could reduce antibiotic resistance and associated burdens.

At present, a big concern arises with counterfeit, adulterated, and substandard drugs. Substandard and counterfeit drugs are the foremost cause of morbidity and mortality [[4], [5], [6]]. As per the World Health Organization report, about 10% of medicines on the global market are counterfeit [7,8]. This scenario is even worse in developing and underdeveloped countries. Post-market surveillance and routine quality assessments are essential in minimizing morbidity and mortality.

Ciprofloxacin is one of the most prescribed fluoroquinolone antibiotics used in the treatment of various infectious diseases, including urinary tract infections, lower respiratory tract infections, bacterial diarrhea, bone and joint infections, skin and soft tissue infections, gonorrhea, and prophylactic in surgery [[9], [10], [11]]. Ciprofloxacin, discovered in 1981 by Bayer, Germany, functions by inhibiting DNA gyrase, type II topoisomerase, and topoisomerase IV, thereby inhibiting cell division. As generic drugs, many pharmaceutical companies in Bangladesh are producing ciprofloxacin preparations.

The prevalence of substandard and or counterfeit medicines, including antibiotics, is incredibly increasing in Bangladesh, especially in the rural areas where the registered physicians are insignificant in numbers, and people are less educated and poor. Mostly quack doctors prescribe medicines in rural areas. Since the use of substandard or counterfeit antibiotics is one of the leading causes of antimicrobial resistance, regular monitoring of marketed brands could give us an insight into their quality. As ciprofloxacin is a widely used antibiotic in Bangladesh, we were interested in evaluating whether the products taken by patients meet the compendial requirements. In our study, we aimed to assess the quality of different commonly used brands of ciprofloxacin 500 mg tablets available in Dhaka and rural areas of Jessore.

## 2 Materials & methods

### 2.1 Chemicals and reagents

Standard Ciprofloxacin hydrochloride was purchased from Sigma-Aldrich (Munich, Germany). *O*-phosphoric acid (85%) and triethylamine (99.5%) were purchased from Scharlau, Spain. HPLC-grade methanol and acetonitrile were obtained from Scharlau, Spain. Other chemicals used in this study were of higher analytical grade and purity.

### 2.2 Collection of different brands of ciprofloxacin tablets

Twenty-two (22) commonly used commercially available brands of ciprofloxacin tablets were collected from different community and model pharmacies in Dhaka & Jessore, Bangladesh. All tablets contain ciprofloxacin as ciprofloxacin hydrochloride form. All of the items' labels claimed that each tablet contained equivalent amount of 500 mg of ciprofloxacin. The samples’ manufacturing license numbers, batch numbers, and production and expiry dates were all double-checked. They were coded as A through V at random and stored at room temperature.

### 2.3 Assay of ciprofloxacin by RP-HPLC method

#### 2.3.1 Preparation of buffer

0.025 M *O*-phosphoric acid was prepared by mixing 1.6 mL of *O*-phosphoric acid (15.7 M) into Milli-Q water to a final volume of 1 L. The pH was adjusted to 3.0 ± 0.1 using triethylamine. The buffer was then filtered using a 0.45 μm membrane filter and degassed.

#### 2.3.2 Preparation of standard and samples

20 mg equivalent of the standard Ciprofloxacin has been dissolved in 40 mL of methanol, buffer mixture (40:60), and mixed properly. Then it was sonicated for 5 min, followed by degassing for another 5 min. Then the preparation was filtered by using a 0.22 μm syringe filter. Different dilutions from this stock solution (0.5 mg/mL) have been made to obtain different concentration standards in the preparation of the standard curve. 10 intact tablets (Ciprofloxacin) from each brand were weighed accurately to obtain the average tablet weight; the tablets were then crushed and triturated in a mortar until a fine powder was obtained. An amount of the powder equivalent to 20 mg ciprofloxacin was weighed accurately and dissolved in 40 mL of the methanol, buffer mixture (40:60), and mixed properly. Then samples were sonicated, degassed, and filtered similarly to the standard ciprofloxacin. 4 μg/mL concentration samples have been prepared by diluting the tablet stock solutions (0.5 mg/mL). 5 μl was injected into RP-HPLC to obtain the retention time & peak area both for standards and tablet samples.

#### 2.3.3 Chromatographic conditions

The High-Performance Liquid Chromatography (HPLC) used in the present study consisted of a Shimadzu LC-20A series instrument equipped with a dual solvent delivery system (LC-20AD). An autosampler injector with a 20 μl sample loop was used to inject the analytes. Chromatographic conditions have been optimized and validated according to the method of Ali et al., 2011 [12]. Chromatographic separation was performed on a reversed-phase C18 column (250 × 4.6 mm; 5 μm particle size) at room temperature for 8 min. Mobile phases (methanol and pH adjusted 0.025 M *O*-phosphoric acid buffer) were mixed at 40:60 ratio, and a flow rate of 1.2 mL/min was maintained. UV detection of the analytes was carried out at 278 nm wavelength. Chromatographic data were collected and processed using Shimadzu LC solution software.

### 2.4 In-vitro dissolution test of ciprofloxacin tablets

#### 2.4.1 Preparation of dissolution buffer & dissolution assay conditions

The dissolution test was attempted utilizing tablet dissolution analyzer USP type 2 (Logan Instruments, USA) in triplicates for each brand. Dissolution media with a concentration of 0.1 N were prepared using hydrochloric acid solution (37%), and the pH was maintained at 1.2. The medium was kept up at 37 ± 0.5 °C. In all the tests, 5 mL of sample was pulled at 0, 5, 15, 30, 45, and 60 min time points from each vessel and replaced with an equal volume of fresh dissolution media to maintain sink condition. Samples were then filtered using a 0.45 μm syringe filter, diluted, and measured by the validated HPLC method.

#### 2.4.2 Determination of drug release kinetics

The dissolution data were analyzed with the DDSolver to calculate mean dissolution time (MDT), similarity factor (F2), and difference. After running several mathematical models, i.e., first-order, Higuchi model, and Korsmeyer-Peppas model, the results collected were then used to decipher the improved gel formulation's drug release mechanism and release rate kinetics in new mathematical models. A model that shows the highest correlation was chosen by comparing R^2^ values. To learn more about release kinetics, DDSolver was used [13].

### 2.5 Assessment of antimicrobial test (zone of inhibition)

#### 2.5.1 Test microorganisms for antibiotic sensitivity test

Bacterial strains were selected based on their clinical importance in causing diseases in humans. The reference test microorganisms used in the study were obtained from HiMedia Labs Pvt. Ltd, India. The microorganisms used were *Escherichia coli* (ATCC 25922), *Staphylococcus aureus* (ATCC 25923), *Pseudomonas aeruginosa* (ATCC 27853), *Salmonella typhi* (6539), and *Shigella flexneri* (ATCC 12022).

#### 2.5.2 Reference standard for antibiotic sensitivity test

Ciprofloxacin was used as a reference standard for antimicrobial susceptibility testing of given bacterial cultures as per the Bauer-Kirby Method. Each standard disc contained 5 μg of ciprofloxacin. Classification of the standard zone of inhibition diameter for different microorganisms is illustrated in Table 1.

**Table 1:** Interpretive criteria for susceptibility categorization used for this study [14].

Microorganism	Standard zone of inhibition diameter (mm)		
	Susceptible	Intermediate	Resistant
*Escherichia coli*	≥21	16–20	≤15
*Staphylococcus aureus*	≥21	16–20	≤15
*Pseudomonas aeruginosa*	≥25	19–24	≤18
*Salmonella typhi*	≥31	21–30	≤20
Shigella flexneri	≥21	16–20	≤12

#### 2.5.3 Preparation of medium & antibiotic discs

Standard discs of Ciprofloxacin 5 μg/disc (HiMedia) were used as a Reference Standard. Preparation of test discs (6 mm in diameter) was punched out from 45 mm Petri Pad (Millipore Corporation, France) and placed in Petri dishes allowing a 2–4 cm distance between each disc, and sterilized in a hot air oven at 160 °C for 1 h.

#### 2.5.4 Preparation of inoculum

1. To prepare the inoculum for the antibiotic sensitivity test, the first broth culture (incubation overnight at 35 °C ± 2) was carried out from a pure culture plate by touching with a sterile loop the tops of each 3 or 4 colonies suspended in Nutrient broth.
2. The broth culture test tube was compared with the turbidity standard and adjusted the density of the test suspension to that of the standard by adding more bacteria. Here, Proper adjustment to the turbidity of the inoculum is essential for ensuring the lawn growth of bacteria which is confluent or almost confluent **[**14**].**

#### 2.5.5 Zone of inhibition determination

1. The plates were inoculated by applying bacterial inoculum to the surface of a large (150 mm diameter) Petri dish containing Sterile Mueller Hinton Agar media.
2. Using a sterile spreader, the bacterial inoculum was spread out all over the surface of the medium three times, rotating the plates clockwise and counter-clockwise through an angle of 60° after each application. Finally, the inoculum was passed around the edge of the agar surface. The agar was left to dry for a few minutes at room temperature with the lid closed. The antibiotic discs, having respective concentrations of our marketed drug (5, 0.5,0.1,0.01,0.001) μg/disc, were placed on the inoculated plates using sterile forceps.
3. The plates were placed in an incubator at 35 °C ± 2 within 30 min of preparation in a CO^2^-free atmosphere.
4. After overnight incubation, the diameter of each zone was measured and recorded in ‘mm’. The zone diameters of each drug are interpreted using the criteria published by the Clinical and Laboratory Standards Institute (CLSI) and European Committee on Antimicrobial Susceptibility Testing (EUCAST) standard [15,16].

### 2.6 Statistical analysis

GraphPad Prism 7.0 was used for the statistical and graphical evaluations. All the results were expressed as mean ± SD. p < 0.05 was considered as significant.

## 3 Results

### 3.1 RP-HPLC method validation for the analysis of ciprofloxacin

**Fig. 1:**
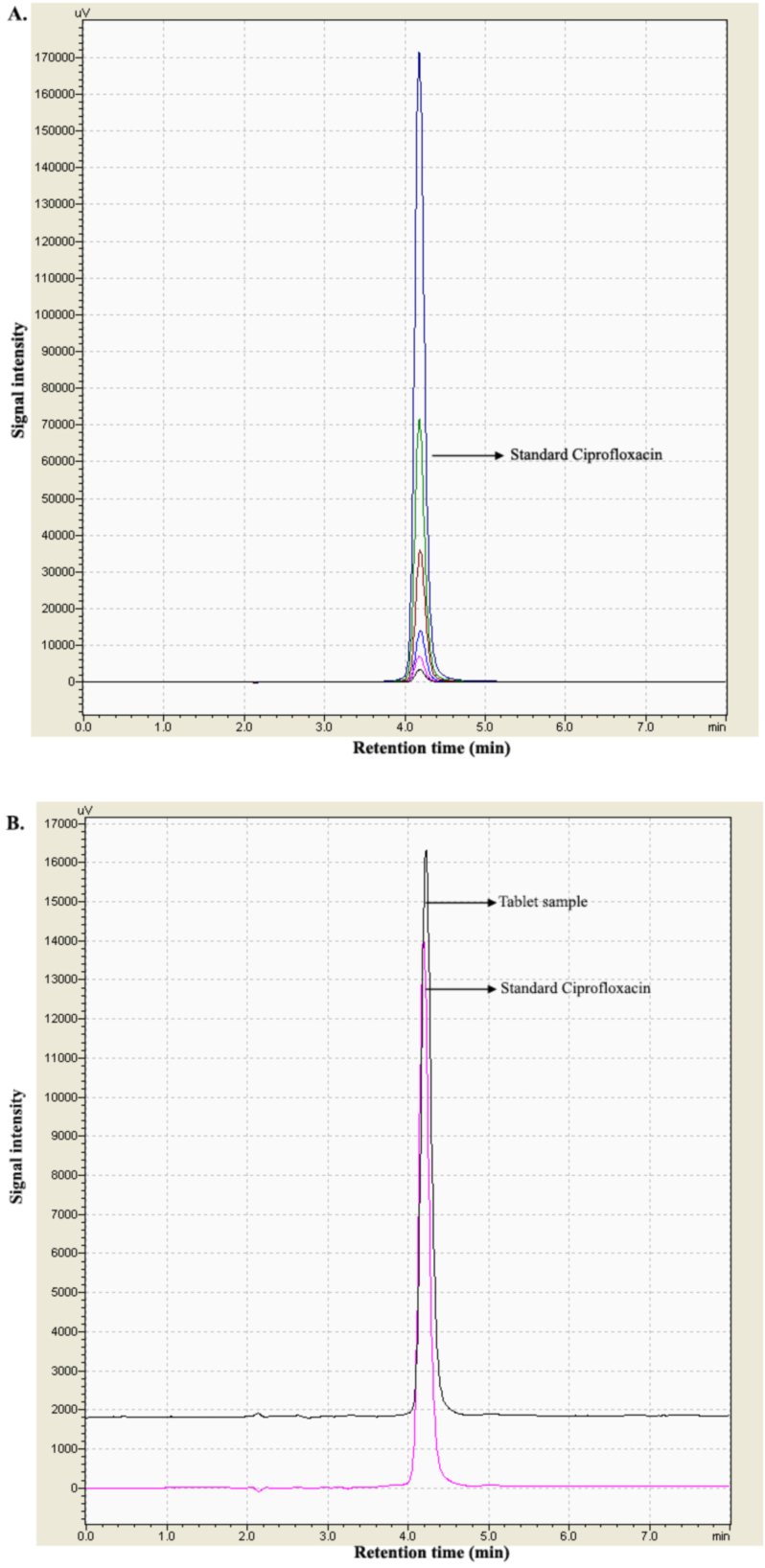
(A) Chromatogram of different concentrations ((1–50) μg/mL) of standard ciprofloxacin samples. (B) Chromatogram of tablet and standard ciprofloxacin samples of 4 μg/mL concentration.

The chromatographic method was validated in terms of system suitability, specificity, precision, accuracy, linearity, LLOQ, carry-over, and stability [17,18]. The theoretical plates were over 2000, and the tailing factor of the chromatographic peak was below 1.2. Fig. 1A showed superimposed chromatograms of different concentration of standard ciprofloxacin samples. The calibration curve of standard ciprofloxacin showed good linearity over the concentration range of (1–50) μg/mL. Fig. 1B demonstrated the superimposed chromatograms of standard ciprofloxacin and tablet sample where the retention time of the desired ciprofloxacin is at 4.2 min.

### 3.2 RP-HPLC analysis of ciprofloxacin from 22 different brands of ciprofloxacin tablets in Bangladesh

**Fig. 2:**
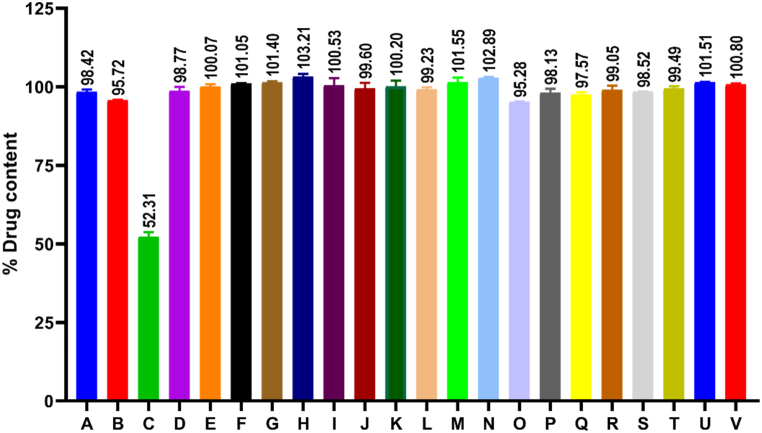
Average % drug content comparing with the declared amount in different brands of ciprofloxacin tablets in Bangladesh.

22 different brands of ciprofloxacin tablets were analyzed using the validated RP-HPLC method. 10 tablets from each of the individual brands were weighed and crushed, and then 4 μg/mL concentration sample was run in RP-HPLC coupled with UV detector. RP-HPLC quantification data showed that 21 out of 22 brands (Fig. 2) complied with the official USP limit (90–110%) and official BP limit (95–105%) of the declared content of the drug in the tablets. Brand C failed to comply with the official USP and BP limit.

### 3.3 Dissolution profiling of 22 different brands of ciprofloxacin tablets in Bangladesh

**Fig. 3:**
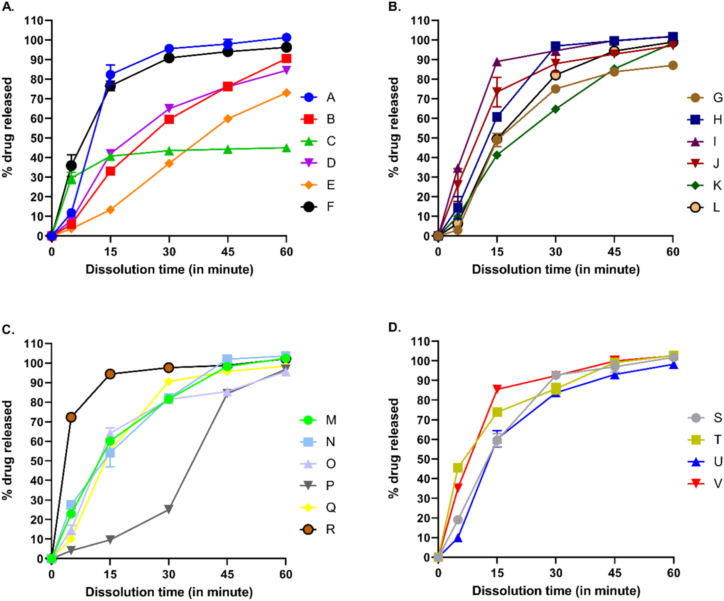
Comparative dissolution profiling of different brands of ciprofloxacin tablets in Bangladesh: Assessing dissolution profiles from brand A-F (A), brand G-L (B), brand M-R (C), and brand S–V (D).

Dissolution is an important parameter in predicting the rate and extent of drug in the systemic circulation. Dissolution test is a key in-vitro quality control test that can predict the bioavailability of an oral drug formulation, including tablets and capsules. As per the USP/NF specification, oral ciprofloxacin tablets should release not less than equivalent to 80% (Q) of the labeled amount within 30 min using USP apparatus type II [19]. According to USP chapter (711) dissolution, at stage 1 (S1), each of the immediate release tablets must release not less than Q+5% (85%) of ciprofloxacin from the labeled amount, whereas at stage 2 (S2), the average release from the tablets must be equal to or greater than Q or 80%, and no unit tablet should release less than Q-15% (65%) of ciprofloxacin [20]. The current study (Table 2) demonstrated that out of 22 brands, 15 brands (brand A, brand F, brand H, brand I, brand J, brand L, brand M, brand N, brand O, brand Q, brand R, brand S, brand T, brand U, and brand V) complied the USP/NF dissolution test specification, where 9 brands (brand A, brand F, brand H, brand I, brand Q, brand R, brand S, brand U, and brand V) followed stage 1 (S1) acceptance criteria of immediate release tablets, and 6 brands (brand J, brand L, brand M, brand N, brand O, and brand T) followed stage 2 (S2) acceptance criteria of immediate release tablets. The other 7 brands (brand B, brand C, brand D, brand E, brand G, brand K, and brand P) failed to comply the USP chapter (711) dissolution acceptance criteria (Fig. 3(A–D)).

**Table 2:** Time-dependent dissolution profiling of different brands of ciprofloxacin tablets in Bangladesh.

Time (min)	Brand A	Brand B	Brand C	Brand D	Brand E	Brand F	Brand G	Brand H	Brand I	Brand J	Brand K	Brand L	Brand M	Brand N	Brand O	Brand P	Brand Q	Brand R	Brand S	Brand T	Brand U	Brand V
**0**	**0**	**0**	**0**	**0**	**0**	**0**	**0**	**0**	**0**	**0**	**0**	**0**	**0**	**0**	**0**	**0**	**0**	**0**	**0**	**0**	**0**	**0**
**5**	**11.80 ± 2.33**	**5.93 ± 0.20**	**29.56 ± 5.00**	**7.92 ± 3.58**	**3.67 ± 0.97**	**35.89 ± 9.43**	**3.05 ± 0.13**	**14.38 ± 9.81**	**34.73 ± 1.47**	**25.96 ± 14.56**	**9.79 ± 0.90**	**6.41 ± 1.07**	**22.96 ± 1.17**	**27.52 ± 3.47**	**14.76 ± 4.23**	**4.11 ± 0.83**	**10.20 ± 1.55**	**72.26 ± 4.09**	**19.09 ± 2.77**	**45.58 ± 1.47**	**9.95 ± 4.11**	**35.17 ± 1.73**
**15**	**82.36 ± 8.54**	**33.10 ± 0.32**	**40.72 ± 2.46**	**42.00 ± 2.84**	**13.33 ± 0.70**	**76.56 ± 4.20**	**48.94 ± 5.89**	**60.74 ± 3.95**	**88.98 ± 1.66**	**73.43 ± 13.07**	**41.31 ± 2.28**	**49.38 ± 2.92**	**60.15 ± 1.42**	**54.11 ± 12.51**	**64.09 ± 4.75**	**9.61 ± 0.90**	**55.33 ± 3.76**	**94.36 ± 0.93**	**59.60 ± 5.78**	**73.87 ± 0.59**	**60.35 ± 7.34**	**85.5 ± 0.27**
**30**	**95.61 ± 2.53**	**59.52 ± 0.54**	**43.58 ± 2.45**	**64.90 ± 3.12**	**37.02 ± 1.24**	**90.90 ± 0.59**	**75.02 ± 2.13**	**96.89 ± 1.83**	**94.51 ± 2.15**	**87.9 ± 7.53**	**64.71 ± 1.52**	**82.12 ± 1.29**	**81.52 ± 0.77**	**82.01 ± 1.63**	**81.40 ± 0.70**	**25.02 ± 1.12**	**90.55 ± 0.32**	**97.62 ± 1.63**	**92.76 ± 1.83**	**85.77 ± 4.66**	**83.73 ± 2.47**	**92.4 ± 1.24**
**45**	**97.92 ± 4.24**	**76.20 ± 0.25**	**44.31 ± 2.34**	**76.13 ± 1.08**	**59.80 ± 1.53**	**94.09 ± 0.68**	**83.77 ± 0.51**	**99.54 ± 1.57**	**99.64 ± 0.53**	**92.81 ± 3.81**	**85.24 ± 1.32**	**94.42 ± 1.52**	**98.24 ± 1.23**	**101.95 ± 0.12**	**85.41 ± 1.43**	**84.41 ± 0.62**	**95.72 ± 1.15**	**98.82 ± 1.44**	**96.98 ± 4.81**	**99.09 ± 1.09**	**93.05 ± 2.02**	**99.94 ± 4.26**
**60**	**101.23 ± 1.00**	**90.52 ± 0.44**	**45.00 ± 1.66**	**84.50 ± 1.36**	**73.05 ± 1.71**	**96.26 ± 1.74**	**87.09 ± 2.43**	**101.69 ± 2.15**	**101.87 ± 1.49**	**97.07 ± 1.65**	**98.37 ± 0.49**	**98.94 ± 0.79**	**102.41 ± 0.74**	**103.66 ± 0.18**	**95.63 ± 0.08**	**96.72 ± 0.84**	**98.45 ± 0.64**	**102.15 ± 1.76**	**101.74 ± 2.53**	**102.68 ± 0.04**	**98.26 ± 3.06**	**102.59 ± 3.80**

Data presented as mean ± SD.

### 3.4 Drug release kinetics from 22 different brands of ciprofloxacin tablets in Bangladesh

Data obtained from the in-vitro dissolution studies (Table-2) has been fitted into various models such as zero order, first order, Hixson-Crowell model, Higuchi model, Korsmeyer-Peppas model, and Weibull model (Table 3) [21,22]. To assess the fit of a model equation, the regression coefficient has been used since it measures the extent to which the regression line represents the data. None of the tablets of 22 different brands follow zero order or Higuchi kinetic models, indicating that drug release from these tablets didn't happen as concentration independent or diffusion-dependent manner (Table 3). Brand F, brand J, brand M, brand R, and brand V followed the first-order kinetic model, which implies that drug dissolution occurs in a concentration-dependent manner. Brand B, brand K, brand M, brand N, and brand S followed the Hixson-Crowell method, which indicates that the tablets of these brands followed dissolution-dependent release kinetics. Brand E, brand R, and brand T followed the Korsmeyer-Peppas kinetic model, where the value of n for brand R and brand T was below 0.45. These indicate that brand R and T followed Fickian diffusion, whereas brand E (n > 1) followed erosion/relaxation dependent drug release mechanism. Almost all the brands followed the Weibull drug release kinetic model (R^2^ value equal to near 0.99). Drug release kinetic data (Table 3) showed that ciprofloxacin tablets of several brands released the drug in combination with multiple dissolution kinetic models.

**Table 3:** Drug release kinetics of 22 different brands of ciprofloxacin tablets in Bangladesh.

Model	Statistics	Brand A	Brand B	Brand C	Brand D	Brand E	Brand F	Brand G	Brand H	Brand I	Brand J	Brand K	Brand L	Brand M	Brand N	Brand O	Brand P	Brand Q	Brand R	Brand S	Brand T	Brand U	Brand V
**Zero-order**	**R**^**2**^	**0.602**	**0.964**	**−0.087**	**0.882**	**0.989**	**0.437**	**0.822**	**0.745**	**0.398**	**0.577**	**0.947**	**0.861**	**0.783**	**0.805**	**0.722**	**0.903**	**0.789**	**−0.206**	**0.752**	**0.464**	**0.786**	**0.444**
	**K0**	**2.161**	**1.649**	**0.998**	**1.640**	**1.240**	**2.076**	**1.771**	**2.136**	**2.205**	**2.047**	**1.823**	**1.981**	**2.071**	**2.099**	**1.928**	**1.553**	**2.039**	**2.256**	**2.102**	**2.145**	**2.000**	**2.197**
	**AIC**	**52.131**	**35.166**	**46.542**	**41.704**	**25.716**	**52.246**	**45.455**	**49.242**	**53.586**	**50.856**	**38.347**	**45.198**	**47.246**	**46.689**	**48.237**	**42.741**	**47.789**	**56.884**	**48.558**	**51.906**	**47.503**	**52.986**
	**MSC**	**0.089**	**2.654**	**−1.782**	**1.397**	**3.926**	**−0.578**	**1.015**	**0.564**	**−0.611**	**−0.141**	**2.222**	**1.260**	**0.640**	**0.743**	**0.425**	**1.840**	**0.794**	**−2.000**	**0.544**	**−0.630**	**0.761**	**−0.534**
**First-order**	**R**^**2**^	**0.941**	**0.982**	**0.219**	**0.989**	**0.953**	**0.995**	**0.964**	**0.967**	**0.985**	**0.990**	**0.980**	**0.967**	**0.993**	**0.983**	**0.976**	**0.790**	**0.969**	**0.997**	**0.984**	**0.981**	**0.975**	**0.990**
	**K1**	**0.080**	**0.031**	**0.015**	**0.033**	**0.018**	**0.090**	**0.040**	**0.066**	**0.110**	**0.075**	**0.038**	**0.049**	**0.061**	**0.060**	**0.055**	**0.023**	**0.056**	**0.251**	**0.064**	**0.096**	**0.055**	**0.104**
	**AIC**	**40.681**	**31.011**	**44.560**	**27.508**	**34.385**	**24.153**	**35.869**	**37.010**	**31.278**	**28.509**	**32.343**	**36.527**	**26.923**	**31.990**	**33.404**	**47.358**	**36.348**	**20.825**	**32.074**	**31.858**	**34.636**	**28.996**
	**MSC**	**1.998**	**3.347**	**−1.452**	**3.763**	**2.481**	**4.104**	**2.613**	**2.603**	**3.107**	**3.584**	**3.222**	**2.705**	**4.027**	**3.193**	**2.897**	**1.071**	**2.701**	**4.010**	**3.291**	**2.712**	**2.906**	**3.465**
**Hixson-Crowell**	**R**^**2**^	**0.955**	**0.993**	**0.122**	**0.980**	**0.969**	**0.975**	**0.958**	**0.985**	**0.967**	**0.981**	**0.994**	**0.983**	**0.995**	**0.991**	**0.963**	**0.827**	**0.983**	**0.746**	**0.995**	**0.953**	**0.980**	**0.976**
	**Khc**	**0.022**	**0.009**	**0.004**	**0.009**	**0.005**	**0.022**	**0.011**	**0.018**	**0.024**	**0.020**	**0.010**	**0.013**	**0.016**	**0.016**	**0.015**	**0.007**	**0.016**	**0.026**	**0.017**	**0.023**	**0.015**	**0.024**
	**AIC**	**39.005**	**24.916**	**45.261**	**30.991**	**31.738**	**33.645**	**36.723**	**32.143**	**36.082**	**32.307**	**25.241**	**32.670**	**24.491**	**28.182**	**36.156**	**46.193**	**32.692**	**47.549**	**24.651**	**37.312**	**33.207**	**34.149**
	**MSC**	**2.277**	**4.363**	**−1.568**	**3.183**	**2.922**	**2.522**	**2.470**	**3.414**	**2.306**	**2.951**	**4.406**	**3.348**	**4.432**	**3.828**	**2.439**	**1.265**	**3.310**	**−0.444**	**4.528**	**1.803**	**3.144**	**2.606**
**Higuchi**	**R**^**2**^	**0.862**	**0.933**	**0.679**	**0.952**	**0.845**	**0.892**	**0.911**	**0.925**	**0.858**	**0.920**	**0.954**	**0.930**	**0.979**	**0.986**	**0.935**	**0.703**	**0.925**	**0.612**	**0.950**	**0.923**	**0.933**	**0.884**
	**Kh**	**14.904**	**10.839**	**7.152**	**10.975**	**7.934**	**14.525**	**11.897**	**14.540**	**15.468**	**14.184**	**12.066**	**13.260**	**14.085**	**14.240**	**13.167**	**9.672**	**13.793**	**16.232**	**14.325**	**14.954**	**13.548**	**15.362**
	**AIC**	**45.767**	**38.901**	**39.224**	**36.335**	**41.476**	**42.313**	**41.315**	**41.874**	**44.929**	**40.852**	**37.473**	**41.120**	**33.229**	**30.778**	**39.548**	**49.451**	**41.599**	**50.077**	**38.975**	**40.294**	**40.536**	**43.586**
	**MSC**	**1.150**	**2.032**	**−0.562**	**2.292**	**1.299**	**1.077**	**1.705**	**1.792**	**0.832**	**1.527**	**2.367**	**1.940**	**2.976**	**3.395**	**1.873**	**0.722**	**1.826**	**−0.866**	**2.141**	**1.306**	**1.922**	**1.033**
**Korsmeyer-Peppas**	**R**^**2**^	**0.868**	**0.986**	**0.987**	**0.966**	**0.991**	**0.956**	**0.921**	**0.925**	**0.926**	**0.943**	**0.988**	**0.945**	**0.979**	**0.986**	**0.935**	**0.947**	**0.928**	**0.991**	**0.950**	**0.993**	**0.935**	**0.943**
	**Kkp**	**18.891**	**3.919**	**25.023**	**6.991**	**0.889**	**28.537**	**8.206**	**14.094**	**31.081**	**21.865**	**5.585**	**8.351**	**14.528**	**14.103**	**14.371**	**0.215**	**11.432**	**62.980**	**14.729**	**30.402**	**11.758**	**29.581**
	**n**	**0.435**	**0.775**	**0.153**	**0.623**	**1.086**	**0.314**	**0.601**	**0.509**	**0.308**	**0.381**	**0.709**	**0.626**	**0.492**	**0.503**	**0.476**	**1.508**	**0.551**	**0.123**	**0.492**	**0.305**	**0.539**	**0.320**
	**AIC**	**47.511**	**31.336**	**21.933**	**36.202**	**26.481**	**38.905**	**42.581**	**43.867**	**42.999**	**40.871**	**31.140**	**41.662**	**35.203**	**32.774**	**41.481**	**41.151**	**43.363**	**29.780**	**40.966**	**27.622**	**42.382**	**41.351**
	**MSC**	**0.859**	**3.293**	**2.320**	**2.314**	**3.799**	**1.645**	**1.494**	**1.460**	**1.153**	**1.523**	**3.423**	**1.850**	**2.647**	**3.062**	**1.551**	**2.105**	**1.531**	**2.517**	**1.809**	**3.418**	**1.615**	**1.406**
**Weibull**	**R**^**2**^	**0.998**	**0.997**	**0.988**	**0.989**	**0.999**	**0.995**	**0.970**	**0.999**	**0.997**	**0.990**	**0.994**	**0.996**	**0.995**	**0.986**	**0.977**	**0.990**	**0.998**	**0.999**	**0.999**	**0.993**	**0.989**	**0.994**
	**b**	**2.388**	**1.287**	**0.200**	**1.041**	**1.557**	**0.926**	**1.179**	**1.713**	**1.470**	**1.065**	**1.280**	**1.527**	**1.122**	**1.116**	**1.052**	**4.346**	**1.633**	**0.699**	**1.390**	**0.760**	**1.353**	**1.275**
	**AIC**	**23.322**	**21.376**	**21.331**	**29.259**	**15.574**	**25.362**	**36.727**	**14.647**	**24.653**	**30.223**	**27.151**	**25.478**	**26.412**	**33.107**	**35.278**	**31.349**	**21.645**	**16.345**	**17.909**	**27.845**	**31.587**	**27.600**
	**MSC**	**4.891**	**4.953**	**2.420**	**3.471**	**5.616**	**3.902**	**2.470**	**6.330**	**4.211**	**3.298**	**4.087**	**4.547**	**4.112**	**3.007**	**2.585**	**3.739**	**5.151**	**4.756**	**5.652**	**3.380**	**3.414**	**3.697**

### 3.5 Fit factor analysis from the dissolution data

For very rapidly dissolving tablets in dissolution test, where 85% of labeled amount of the drug is released within 15 min, a dissolution profile comparison in between test and the reference product is not needed. Dissolution profiles of tablets of different brands have been compared using a model-independent approach of difference factor (f1) and similarity factor (f2). Two dissolution profiles are considered similar and bioequivalent when the f1 value is between 0 and 15, and the f2 value is between 50 and 100 [23]. Dissolution data of brand I, brand R, and brand V at 15 min showed that average ciprofloxacin release from these tablets were more than 85% of the claimed amount. Dissolution data of all other brands were compared with the innovator equivalent tablet brand S (Table 4). Fit factor analysis data showed that 10 brands (Brand A, brand F, brand H, brand J, brand L, brand M, brand N, brand O, brand Q, and brand U) exhibited similar dissolution profiles as innovator equivalent, S, whereas 8 brands (Brand B, brand C, brand D, brand E, brand G, brand K, brand P, and brand T) failed to comply.

**Table 4:** Fit factor analysis of different brands of ciprofloxacin tablets in Bangladesh.

Statistics	A/S	B/S	C/S	D/S	E/S	F/S	G/S	H/S	I/S	J/S	K/S	L/S	M/S	N/S	O/S	P/S	Q/S	R/S	S/S	T/S	U/S	V/S
**f1**	**9.279**	**28.335**	**50.760**	**25.594**	**49.515**	**11.650**	**19.529**	**3.403**	**13.382**	**9.297**	**19.113**	**10.513**	**4.746**	**8.525**	**10.232**	**40.599**	**5.381**	**25.669**	**0**	**13.719**	**7.120**	**12.463**
**f2**	**50.239**	**34.317**	**20.739**	**37.203**	**22.295**	**50.204**	**43.609**	**76.322**	**43.186**	**57.254**	**41.090**	**54.426**	**65.084**	**59.450**	**55.993**	**22.576**	**67.427**	**29.216**	**100**	**44.839**	**61.954**	**45.072**

f1 = difference factor; f2 = similarity factor.

### 3.6 Assessment of antimicrobial activity by Kirby-Baeur disc diffusion susceptibility test

**Fig. 4:**
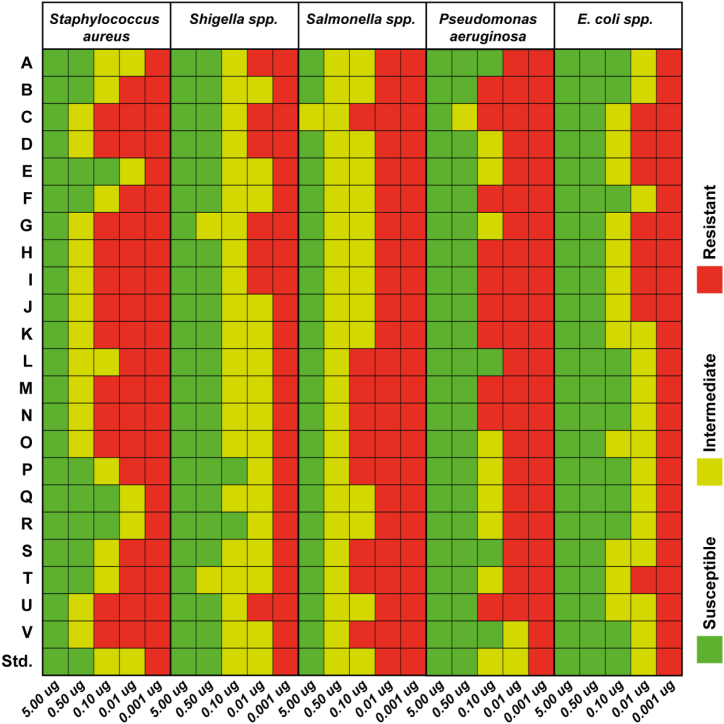
Comparative analysis of the activity of different brands of ciprofloxacin tablets against 5 different standard microorganisms.

Antibiotic disc of 22 different brands of ciprofloxacin tablets has been prepared in aseptic condition using different amount of the drug (5 μg, 0.5 μg, 0.1 μg, 0.01 μg, and 0.001 μg). Then these discs were placed on the inoculated plates and incubated overnight to measure the zone of inhibition. Disc diffusion data (Fig. 4) showed that 5 μg disc of all 22 brands of ciprofloxacin tablets exhibited good susceptibility against all the test microorganisms (*Escherichia coli*, *Staphylococcus aureus*, *Pseudomonas aeruginosa*, *Salmonella typhi*, and *Shigella flexneri*). Except for brand C, all other brand's ciprofloxacin discs showed intermediate susceptibility against all the test microorganisms, even at lower amounts. These results comply with the RP-HPLC results, which showed that the ciprofloxacin content in brand C was 51.51% of the labeled amount.

## 4 Discussion

Generic drugs are medications with the same qualitative and quantitative composition of active pharmaceutical ingredient(s) whose therapeutic efficacy has been determined and compared with the reference drug using bioequivalence and pharmacoequivalence studies [24,25]. In many healthcare systems, generic drugs have been introduced to ensure access to medication at affordable prices in comparison with the innovator brand's drug. Although World Health Organization (WHO) emphasizes the use of generic drugs to reduce medicine cost burden & increase access to treatment, it is also very important that the generics are of similar strength, quality, and efficacy. According to WHO report, about 10% of the medicines on the global market are counterfeit [7,26], and the situation is greatly alarming for developing and underdeveloped countries [27,28].

Although the qualities of drugs are regularly checked during production (in process), and after production by the pharmaceutical companies, it is equally important to check the safety, potency, and efficacy of the marketed products by regulatory bodies and researchers to minimize the availability of substandard, counterfeit, adulterated, and spurious products in the market. Failure to ensure the quality of the finished products in the market can be devastating, especially for drugs like antibiotics, which might result in antibiotic resistance with increased morbidity and mortality [29,30]. Antibiotic resistance is not only a national threat but rather a threat to the global health system. Bangladesh, a developing country in Asia, demonstrated higher antibiotic resistance in recent years resulting in increased mortality [3,31,32]. Routine quality assessment, post-market surveillance, ensuring rational drug use, and enacting regulatory guidelines are essential in minimizing morbidity and mortality.

As part of the quality control check, it is important to ensure that the generic drugs are pharmaceutically equivalent and bioequivalent with the innovator drugs. Dissolution studies of solid dosage forms (i.e., tablets, capsules, etc.) are performed to examine the rate and extent of drugs into the solution, and dissolution tests are conducted to predict the availability of a drug in the systemic circulation. It is equally important to examine whether data obtained from dissolution and other quality control studies follow regulatory acceptance guidelines (i.e., USP, BP, etc.) [[33], [34], [35]].

The average % potency of 22 different brands of ciprofloxacin tablets in Bangladesh has been presented in Fig. 2. The present study showed that 95.45% of brands (21 out of 22 brands) had complied official USP limit (90–110%) and official BP limit (95–105%) of ciprofloxacin content to the labeled amount (500 mg). Brand C tablets have an average % potency (51.51 ± 0.97) of the labeled amount, which clearly failed to follow the official USP and BP limit.

Dissolution profiles of 22 different brands of ciprofloxacin tablets in Bangladesh have been presented in Table 2 and Fig. 3(A–D). As per the USP/NF specification, oral ciprofloxacin tablets should release not less than equivalent to 80% of the labeled amount within 30 min using USP apparatus type II [18]. The present study exhibited that 15 brands out of 22 followed USP chapter (711) dissolution test specification, where 9 brand (brand A, brand F, brand H, brand I, brand Q, brand R, brand S, brand U, and brand V) followed stage 1 (S1) acceptance criteria of immediate release tablets (individual unit released at least 85% drug of the labeled amount) and 6 brand (brand J, brand L, brand M, brand N, brand O, and brand T) followed stage 2 (S2) acceptance criteria of immediate release tablets (average drug release from tablets were at least 80% or more of the labeled amount, and no individual tablet released less than 65%). The remaining 7 brands (brand B, brand C, brand D, brand E, brand G, brand K, and brand P) failed to comply USP/NP dissolution test acceptance criteria (Table 2). The decreased availability of ciprofloxacin from these 7 brands after 30 min could be due to variations in nature, composition, and quantity of excipients used in the formulation, together with compounding process or instrumental variations (i.e., granulation, compression, etc.).

To know which release mechanisms tablets follow to disseminate drugs into the solution, various model-dependent kinetic approaches have been fitted into the dissolution data of 22 different brands of ciprofloxacin tablets in Bangladesh (Table 3). Among the 6 kinetic models (zero order, first order, Hixson-Crowell model, Higuchi model, Korsmeyer-Peppas model, and Weibull model), the model(s) that best fit the dissolution data will provide high value for correlation coefficient (R^2^). Table 3 data showed that all the brands followed the Weibull drug release kinetic model (R^2^ value near or equal to 0.99). We also found that Brand F, brand J, brand M, brand R, and brand V followed the concentration-dependent first-order release kinetic model, whereas Brand B, brand K, brand M, brand N, and brand S followed the Hixson-Crowell method. Brand R and brand T (n < 0.45) followed Fickian diffusion, whereas brand E (n > 1) followed erosion/relaxation dependent drug release mechanism. Drug release kinetic data showed that several brands released drugs in combination with multiple dissolution kinetic models.

In this study, model-independent methods (f1 and f2 factors) have been performed in comparison with the innovator equivalent, brand S [36], and presented in Table 4. According to FDA, two dissolution profiles are considered similar and bioequivalent when the f1 value is between 0 and 15, and the f2 value is between 50 and 100 [21]. Dissolution profiles of brand I, brand R, and brand V showed that these tablets released more than 85% of claimed amount of ciprofloxacin within 15 min in the dissolution test, and therefore profile comparison with the innovator equivalent, brand S were not required. Table 4 shows that 10 brands (Brand A, brand F, brand H, brand J, brand L, brand M, brand N, brand O, brand Q, and brand U) exhibited similar dissolution profiles as innovator equivalent, S, whereas 8 brands (brand B, brand C, brand D, brand E, brand G, brand K, brand P, and brand T) failed to comply similar dissolution profiles as the reference brand.

Finally, the antimicrobial activity of these 22 brands of ciprofloxacin tablets has been assessed using 5 different drug quantities against various known laboratory standard microorganisms (*Escherichia coli*, *Staphylococcus aureus*, *Pseudomonas aeruginosa*, *Salmonella typhi*, and *Shigella flexneri*) (Fig. 4). 5 μg disc of all 22 brand ciprofloxacin tablets exhibited good susceptibility against all the test microorganisms in Kirby Baeur disc diffusion test. All brands, except C, showed intermediate susceptibility against all the test microorganisms, even at lower amounts. These results are compliant with the RP-HPLC data, which showed that the content of the ciprofloxacin in brand C was significantly lower than the labeled amount.

## 5 Conclusions

The current study evaluated the quality attributes of 22 different brands of ciprofloxacin tablets in Bangladesh. 95.45% of brands (21 out of 22 brands) showed compliance with USP & BP specification of drug content when analyzed directly from crushed tablets. Dissolution studies showed that only 68.2% of brands (15 out of 22) followed USP/NF dissolution test specifications, whereas 31.8% failed to comply. Although most drugs followed Weibull drug release kinetics, several brand tablets followed multiple release kinetics. Fit factor analysis showed that 8 brands out of 22 (36.4%) failed to comply similar dissolution profiles with the reference product. And finally, in the Kirby-Baeur disc diffusion test, 5 μg discs of all brands showed good antimicrobial sensitivity against 5 different known laboratory standard microorganisms. Considering all data from the current study, we found that 63.6% of brands (14 out of 22 ciprofloxacin tablet brands) complied with all the guidelines for USP and BP specified drug content, USP/NF specified dissolution criteria, FDA specified similarity and dissimilarity factors, and good antimicrobial sensitivity against known laboratory standard microorganisms.

## Author contribution statement

Md. Hasanuzzaman Shohag: Performed the experiments; Analyzed and interpreted the data; Wrote the paper. Syed Abdul Kuddus; Esfat M. Saim Brishty; Salman Sakir Chowdhury; Md. Tofazal Hossain; Maruf Hasan; Sabrin Islam Khan: Performed the experiments. Murad Hossain: Analyzed and interpreted the data. Hasan Mahmud Reza: Conceived and designed the experiments; Analyzed and interpreted the data; Contributed reagents, materials, analysis tools or data; Wrote the paper.

## Data availability statement

Data included in article/supplementary material/referenced in article.

## Declaration of competing interest

The authors declare that they have no known competing financial interests or personal relationships that could have appeared to influence the work reported in this paper.
